# Comparative Analysis of the Effects of Various Storage Packages on Maize (*Zea mays* L.) Postharvest Quality

**DOI:** 10.1155/ijfo/3143021

**Published:** 2026-09-15

**Authors:** Paa Kwesi Bordoh, Evans Ntim Amedor, Kombat Damlun, Evans Frimpong Boateng

**Affiliations:** ^1^ Department of Horticulture and Crop Production, School of Agriculture and Technology, University of Energy and Natural Resources, Sunyani, Ghana, uenr.edu.gh; ^2^ Department of Food Science and Technology, School of Agriculture and Technology, University of Energy and Natural Resources, Sunyani, Ghana, uenr.edu.gh

**Keywords:** aflatoxin, insect damage, postharvest, storage bag, Tain

## Abstract

Maize is a critical staple crop, yet postharvest losses largely due to inadequate storage packages remain a significant issue in Tain District, Bono Region, Ghana. In the present study, different storage packages (treatments): hermetic bags, nylon sacks, and jute sacks (all stacked on wooden pallets) compared with unpackaged maize (maize stored on a lightweight corn tarp elevated on a wooden pallet) effect on postharvest quality of maize was investigated to address critical postharvest losses that threaten food security. Laboratory analysis examined insect damage, physical and nutritional quality and aflatoxin levels. Results showed significant differences (*p* < 0.05) across all parameters, with hermetic bags proving most effective in reducing insect‐damaged kernels, maintaining stable moisture content and preserving protein, fibre and carbohydrate levels while minimising aflatoxin accumulation. Nylon sacks performed moderately well but were less effective than hermetic bags in preserving nutritional content and preventing grain weight loss. Jute sacks and the control group showed poor performance with higher grain damage, moisture fluctuations, discolouration, weight loss and nutrient degradation. Critically, aflatoxin levels in jute sacks and control samples reached 28.36 and 42.38 ppb, respectively, exceeding Ghana′s maximum safety permissible limit of 15 ppb, whereas hermetic bags and nylon sacks maintained significantly lower levels. The findings underscore the critical role of hermetic bag storage as the most reliable option in reducing postharvest losses. Yet, the nylon sacks could be used, especially by the underprivileged or financially constrained stakeholders in the maize value chain, for short to medium‐term storage.

## 1. Introduction

Maize (*Zea mays* L.) belongs to the grass family (Gramineae) and serves as a crucial food crop for both humans and livestock, providing energy, vitamins and minimal amounts of protein [1, 2]. As a staple crop cultivated throughout Ghana, maize is the primary carbohydrate source in most Ghanaian meals. It has recently surpassed cassava as Africa′s most important food crop in terms of calories consumed [3] and serves as a primary income source for producers in maize surplus regions. In Ghana, maize is the largest consumed staple crop and forms the foundation of many traditional food preparations, including ‘banku’, ‘kenkey’ and ‘tuo zaafi’ [4]. Cultivated across all 16 regions of the country, with the Ashanti, Eastern, Bono, Central and Bono East regions as the key maize‐producing areas [5]. Maize production is important as root and tuber agricultural staple crops, vital as poultry feed and integral for the brewing industry. Maize is closely associated with household food security; low‐income households are considered food insecure if they lack maize stock, regardless of other available foods. The crop accounts for 50%–60% of Ghana′s total cereal production [6]. The USDA Foreign Agricultural Service (FAS) Accra forecasts Ghana′s maize production 2026/2027 (July to June) at 3 million metric tons, reflecting an upward trajectory in agricultural yields [7]. With Ghana achieving more than 50% of the total cereal production and approximately 3.3% of total agricultural production [8], proper packaging and storage practises are essential to prevent losses, maintain postharvest quality and mitigate acute food insecurity. According to Zacharia et al. [9], food loss is more relevant than food waste in sub‐Saharan Africa. The United Nations estimates that 1.3 billion tons of food are lost globally each year [10] and in the food supply system, 13.3% of the world′s food is lost or wasted [11], highlighting the critical importance of effective storage packaging in postharvest handling. According to Kwadzokpui et al. [12] and Adams et al. [13] in Ghana, postharvest processes result in the loss of 18% of maize. Additionally, Ghanaian maize growers lose $64.70 per acre. Consequently, postharvest storage packaging significantly affects agricultural product quality and marketability, playing a vital role in food storage between harvests, nutrient preservation and the distribution and export of agricultural produce [14]. The economic importance of packaging depends primarily on the food industry′s status, the stockpiling economy′s existence and expected harvest volumes [15]. Modern technological advances and increased consumer convenience demands have led to numerous new storage packaging options, necessitating research into food‐storage package material interactions. Effective storage packaging conditions preserves postharvest grain quality by preventing deterioration of the food grain while maintaining optimal moisture, temperature and humidity levels for storage [16]. Research by Rajapakshe et al. [17] establishes that poor packaging can lead to diseases and pest attacks, affecting produce market value. Odjo et al. [18] and Baributsa and Baoua [19] empirically demonstrated that hermetic bags maintained maize and soybean seed quality while minimising grain quality loss. On the other hand, wet maize kept in hermetic storage for a month or more has been shown to lose quality [20]. To lower the moisture content of maize grains, farmers in the Tain area of Ghana, a humid tropical nation, mostly rely on sun drying the grain in the field and continuing at the house. However, during storage, conventional techniques are used to manage grain dryness due to unfavourable weather conditions (rain or cloud cover) and prevent insect pests. Poor storage packaging and inadequate storage conditions facilitate insect infestation and mould growth, leading to mycotoxin development that compromises quality and increases losses. In most maize‐growing areas, like the Tain district in Ghana, smallholder farmers predominantly use jute or nylon sacks as packaging materials due to their accessibility, affordability and ease of handling. However, limited information exists regarding how these storage bags affect maize postharvest quality. Furthermore, there is no proof that storage technique affects storage and postharvest storage losses in the Tain district to the best of our knowledge. Therefore, this study is aimed at investigating the comparative effects of different storage packages on postharvest quality of maize, specifically on insect pest damage levels, physical quality (moisture, discolouration, grain weight loss), nutritional quality (protein, fibre and carbohydrate content) and aflatoxin levels. This would add to the already existing literature on informed choices in choosing the best packages for short‐ to medium‐term storage of stored grain to reduce postharvest losses and ensure food security of stored grains.

## 2. Materials and Methods

### 2.1. Materials

The ‘Omankwa’ variety of maize was obtained from a local farm when harvested 90–95 days after sowing and the storage packaging materials were procured from the open market in the Tain District of Ghana. All chemicals and reagents used were of analytical grade and supplied by the pilot laboratory.

### 2.2. Study Area

**Figure 1:**
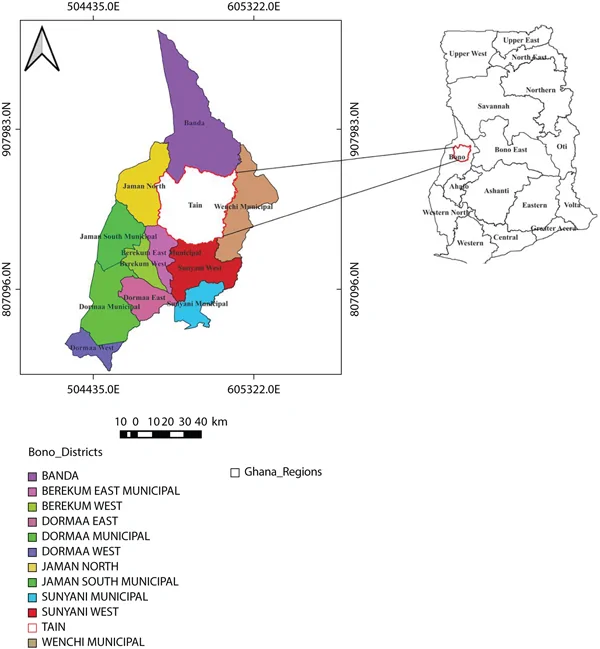
Map of Ghana showing the study area (Tain District, Bono Region).

The study was conducted in the Tain District of the Bono Region, Ghana. Approximately 115,568 people live in the district, with more than 60% of them involved in farming [21]. The Tain District experiences year‐round high temperatures, with an average of roughly 24.5°C. The average temperature ranges from 21.2°C to 30.9°C. February, March and April are the hottest months. There are two rainy seasons in a year due to the bimodal nature of the rainfall pattern. Rainfall peaks in June during the major season, which runs from April to July, and in October during the minor season, which runs from mid‐August to mid‐November. August has a little dry spell, but the main one lasts from November to March. Rainfall averages between 1140 and 1270 mm per year [22, 23]. The district is one of the major maize‐producing areas in the middle belt of Ghana. As an area of interest for its production capabilities, data gathered by Ministry of Food and Agriculture (MoFA) and African Postharvest Losses Information System (APHLIS) have established that postharvest loss (PHL) is a significant challenge in the area [22, 24]. The district is located Northwest of Sunyani (regional capital), positioned between latitudes 7°12 ^′^ and 8°45 ^′^ North and longitudes 2°52 ^′^ West and 0°28 ^′^ East. Tain District covers a land area of 4125 square kilometres. The district is bounded by Wenchi Municipality to the east, Jaman North to the west, Sunyani West to the south and Berekum Municipality to the southwest [21] (Figure 1). ‘Omankwa’ is the most common maize variety grown in the area.

### 2.3. Sample Collection, Preparation and Treatment

Freshly harvested maize ‘Omankwa’ variety was sampled, the husk or outer sheath was torn off from the cob, thoroughly dried (sun or open‐air drying) to standard moisture content (12%–13%), shelled or threshed (a power (motorised) maize shelling machine), winnowed and cleaned of unwanted materials (stray seeds, stones, dirt or sands, metals, straws, leaves, stems, cob pieces, damaged kernels, discoloured grains, weeds). About 10 kg of a cleaned and properly dried maize was sampled and kept in each storage bag or package (treatment) and tightly secured with a thread. The maize grains were subjected to treatments (stored packages) comprised of a jute sack measuring 112 × 68 cm, a nylon sack measuring 200 × 75 cm and a hermetic (super grain) bag measuring 137 × 70 cm all stacked on wooden pallets. Maize kept on a lightweight corn tarp elevated on a wooden pallet on the warehouse floor, however, served as the control. All the experimental samples were stored for a period of 5 months with an average ambient relative humidity of 60%–65% and temperature ranging between ±18°C to ±20°C approximately by utilising a digital thermo‐hygrometer (Hanna, H18564, Italy). Samples were taken monthly and for each month, 12 observations (three for each treatment) were considered for physical properties, nutritional composition and aflatoxin content evaluation at the Kwame Nkrumah University of Science and Technology (KNUST) Central Laboratory. A randomised complete block design (RCBD) with three replicates per treatment was employed and sampling of maize per storage package was conducted randomly using a probe (Stainless Steel 304 Grain Sampler, China). The four different treatment groups were denoted as T1 (control), T2 (jute bag), T3 (hermetic bag) and T4 (nylon sack). In addition, the four different treatment groups were subjected to storage time denoted as D0 (Day 1), M1 (1 month), M2 (2 months), M3 (3 months), M4 (4 months) and M5 (5 months).

### 2.4. Evaluation of Grain Damage by Insect Pests

Insect damage to grains was assessed using a combination of counting and weighing methods. Each month, 500 g of maize grains was sampled from each treatment using a probe (Stainless Steel 304 Grain Sampler, China), which was inserted from the centre and the four cardinal points [25]. The number of insect‐damaged and undamaged grains was identified using a hand lens (Belomo 10× magnification, Belarus) to search for the presence of holes in the kernel [26]. Insect‐damaged kernels (IDK) were weighed. The percentage of IDK was calculated by number (% (IDK_nb_) and by weight (% (IDK_wb_) using the Equations (1) and (2) [27].

(1) Percent IDK by number=number of IDKtotal number of kernels×100,

(2) Percent IDK by number=weight of IDK500×100,

where IDK represent insect‐damaged kernels.

### 2.5. Physical Quality Assessment

#### 2.5.1. Moisture Content

The moisture content was estimated following Asres et al. [28] methodology with moderation. Crucibles were oven‐dried at 180°C for 1 h to a constant weight, then cooled in a desiccator to room temperature (designated as W1). About 5 g of maize samples from each packaging material were placed in crucibles monthly and reweighed (W2). Crucibles were heated in a hot‐air oven at 115°C for 3 h, transferred to a desiccator, cooled to room temperature and weighed (W3).

(3) Moisture content %=initial weight−final dry weightinitial weight×100.

#### 2.5.2. Discolouration

A 100 g sample from each treatment was examined under normal light, and discoloured grains were separated from normal grains. When a grain has a colour other than the normal colour of maize, it is considered discoloured. This procedure was replicated three times for each treatment, allowing for the calculation of percentage discolouration and mean values based on Equation (4).

(4) Discolored grain %=number of discolored graintotal numbers of grain×100.

#### 2.5.3. Grain Weight Loss

The conventional count and weigh or gravimetric method employed for the grain weight loss due to biodegradation reported by Manu et al. [27] was used based on Equation (5).

(5) Weight loss %=Wu×Nd−Wd×NuWu×Nd+Nu×100,

where Wu is the weight of undamaged grains, Nu is the number of undamaged grains, Wd is the weight of damaged grains and Nd is the number of damaged grains.

### 2.6. Nutritional Quality Assessment

#### 2.6.1. Protein Content

The standard method of the Association of Official Analytical Chemists (AOAC) [29] and Hamisu et al. [30] was used to evaluate the protein content. The analysis was carried out in triplicate. Briefly, the Kjeldahl method following the sample preparation, digestion, distillation, titration and calculation was used to determine the crude protein. The measured nitrogen was multiplied by a conversion factor of 6.25.

#### 2.6.2. Crude Fibre Content

A 2 g sample was transferred to a 9‐cm filter paper on a 60° funnel, extracted with three 25 mL ether portions under vacuum until dry, then moved to a 600 mL beaker. The sample was treated with 20‐mL ceramic fibre suspension, 200 mL boiling 1.25% sulfuric acid (H_2_SO_4_) and antifoam, and then boiled for 30 min with periodic rotation. Contents were filtered through a Buchner funnel precoated with ceramic fibre, rinsed with boiling water portions and dried. The fibre mat was returned to the beaker with 200 mL of boiling 1.25% sodium hydroxide (NaOH) and boiled for 30 min. After filtering and washing with sulfuric acid (H_2_SO_4_), water (H_2_O) and alcohol, the mat and residue were transferred to an ashing dish, dried at 130^°^C ± 2^°^C for 2 h, cooled, weighed and ignited at 600^°^C ± 15^°^C to constant weight. A blank determination was performed using identical quantities of fibre, acid and alkali by employing Equation (6) [31].

(6) %Crude fiber d.b= dry residues Wt.g ignited residue Wt.g blank Wt.loss gsample Wt.g×sample moisture %×100,

where d.b is the dry basis and Wt. is the weight.

#### 2.6.3. Carbohydrate Content

The phenol‐sulfuric acid method as described by Khan et al. [32] was used to estimate carbohydrate content. The powder of each treatment (0.1 g) was mixed in 5 mL of 2.5 N hydrochloric acid (HCl) and heated in a water bath for 3 h. It was then neutralised by adding sodium carbonate (Na_2_CO_3_), and the volume was increased to 100 mL. Glucose (C_6_H_12_O_6_) was used as a standard for carbohydrate estimation. Each experimental sample was mixed with 1 mL of 5% phenol solution and 5 mL of 96% sulfuric acid (H₂SO₄) solution and kept at 30°C for 20 min. After that, absorbance was taken at 490 nm with a UV‐vis spectrophotometer (TU‐1810; Purkinje, General Instrumentation Co. Ltd., Beijing, China), and the linear regression equation obtained from the standard curve was used to estimate carbohydrate in the maize sample.

### 2.7. Aflatoxin Levels Evaluation

Laboratory analysis and protocols for aflatoxin levels were adopted from Agbetiameh et al. [33] with modification. In brief, aflatoxin was extracted from maize in Month 3 and Month 5 by combining 20 g of ground samples with 100 mL of 70% methanol (CH_3_OH). The suspension was centrifuged for 30 min at 400 rpm and filtered through Whatman Grade 1 filter paper. Filtrates were collected in 250‐mL separatory funnels, combined with 100‐mL distilled water and extracted twice with 25‐mL methylene chloride (CH_2_Cl_2_). The methylene chloride (CH_2_Cl_2_) phase was filtered through a bed of 25‐g anhydrous sodium sulphate (Na_2_SO_4_) contained in fluted Whatman Grade 4 filter paper, combined and evaporated to dryness in a fume hood. Residues were then dissolved in 1 mL of methylene chloride (CH_2_Cl_2_) and subjected to scanning densitometry. Homogenates were directly spotted (4 *μ*L) alongside aflatoxin standards on a thin‐layer chromatography (TLC) aluminium (20 × 10 cm) silica gel 60 F_254_ plates and developed with diethyl ether‐methanol‐water (C_4_H_10_O: CH_3_OH: H2O, 96: 3:1). Plates were visualised under ultraviolet light (365 nm) for the presence or absence of aflatoxins. Aflatoxins were quantified directly on TLC plates with a scanning densitometer and quantification software. The limit of detection (LOD) and quantification (LOQ) were 0.19 and 0.6 *μ*g/kg. The following concentrations of 0.25, 1.25, 12.5, 25 and 62.5 *μ*g/kg were used as validation bases on the linearity of calibration curve with correlation coefficient of *R* ^2^ = 0.996. The recovery was estimated spiking blank at concentrations of 1, 5 and 10 *μ*g/kg which showed an acceptable range of 70%–110% with the procedure expressed as relative standard deviation (RSD), < 10% [34, 35].

### 2.8. Statistical Analysis

All data were analysed using the OriginLab software (2026b; Version 10.35, Origin Lab Corporation, Northampton, Massachusetts, United States). A one‐way analysis of variance (ANOVA) and Tukey′s test was applied to find which specific group means were different from each other (pairwise checks) to evaluate significant differences at (*p* < 0.05). Analysis was carried out in three replications, and the data values were expressed as mean ± standard deviations (*n* = 3).

## 3. Results and Discussion

### 3.1. Level of Grain Damage by Insect Pests

**Figure 2:**
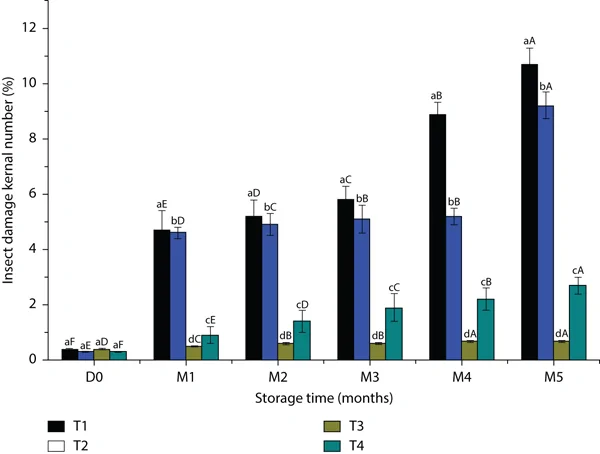
Observed impact of postharvest storage bags on insect damage kernel number during 5 months of storage. Values are means ± standard deviation (*n* = 3); different lowercase letters indicate significant differences (*p* < 0.05) across the treatment (T1–T4), whereas different upper letters indicate significant differences (*p* < 0.05) across the storage time (D0‐M5)—based on Tukey′s test. T1 (control); T2 (jute bag); T3 (hermetic bag); T4 (nylon sack); D0 (Day 1); M1 (1 month); M2 (2 months); M3 (3 months); M4 (4 months) and M5 (5 months).

**Figure 3:**
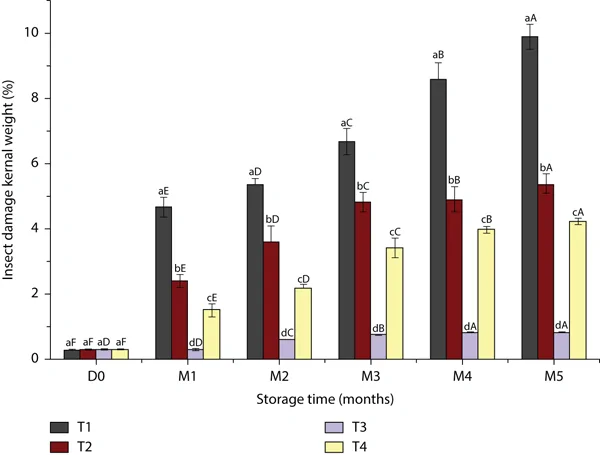
Observed impact of postharvest storage bags on insect damage kernel weight during 5 months of storage. Values are means ± standard deviation (*n* = 3); different lowercase letters indicate significant differences (*p* < 0.05) across the treatment (T1–T4), whereas different upper letters indicate significant differences (*p* < 0.05) across the storage time (D0‐M5)—based on Tukey′s test. T1 (control); T2 (jute bag); T3 (hermetic bag); T4 (nylon sack); D0 (Day 1); M1(1 month); M2 (2 months); M3 (3 months); M4 (4 months) and M5 (5 months).

A significant difference (*p* < 0.05) of treatments (T1, T2, T3 and T4) and storage time (months) on grain damage by insect pests was observed. Grain damage increased progressively over time, becoming most pronounced by the end of the 5th month in T1, T2 and T4 samples, except T3 samples, which showed no damage (Figures 2 and 3). The average percentage of IDK across all storage packages and sampling periods was less in terms of number (IDK_nb_) compared with weight (IDK_wb_). The IDK_nb_ and IDK_wb_ values increasing trend were generally consistent across months, and where variations occurred, they followed a predictable trend of increasing over time.

The study also observed variations in insect damage levels across the different storage packages. Specifically, the T3 and T4 treated samples exhibited relatively low levels of kernel damage; however, this was more pronounced in T3 samples comparatively, suggesting that T3 was effective in inhibiting insect infestations of the maize. The outcomes are consistent with research by Baoua et al. [36], who showed that Purdue improved crop storage (PICS) hermetic storage greatly reduced insect damage to grains that were stored. In contrast, samples, especially in the control and jute sacks, demonstrated relatively high levels of kernel damage. According to Misra et al. [37], traditional storage materials like jute sacks fail to provide effective barriers against the fundamental biological, chemical and physical processes that lead to grain quality loss. Grain‐filled hermetic bags have the potential to produce self‐regulating environments for metabolic activities with lower oxygen levels and higher carbon dioxide levels.

Misra et al. [37] state that microbial deterioration of maize is influenced by temperature, relative humidity of the storage environment, microbial cross contamination during harvest or storage, grain moisture content and gas composition. As a result, the results of this study show that T3 considerably decreased insect metabolic activity by providing the maize grains with an airtight, regulated environment. It is important to understand that insects use respiration to increase carbon dioxide and decrease available oxygen. Consequently, insect metabolism is significantly slowed. Because the grains were well dried and cleaned, there was less oxygen depletion and carbon dioxide accumulation, indicating the high rate of metabolic activity in the hermetic bags. As a result, T3 samples experienced altered fecundity, delayed insect development and poor metamorphosis consistent with similar findings reported by Ognakossan et al. [38].

### 3.2. The Effect of Postharvest Storage Bags on the Quality of Maize

#### 3.2.1. Moisture Content

**Figure 4:**
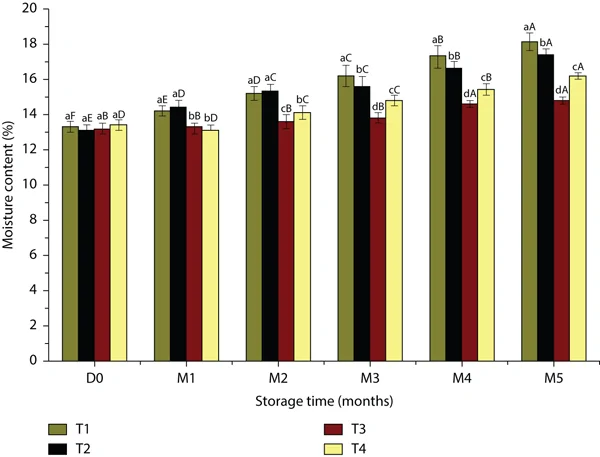
Observed impact of postharvest storage bags on maize moisture content during 5 months of storage. Values are means ± standard deviation (*n* = 3); different lowercase letters indicate significant differences (*p* < 0.05) across the treatment (T1–T4), whereas different upper letters indicate significant differences (*p* < 0.05) across the storage time (D0‐M5)—based on Tukey′s test. T1 (control); T2 (jute bag); T3 (hermetic bag); T4 (nylon sack); D0 (Day 1); M1 (1 month); M2 (2 months); M3 (3 months); M4 (4 months) and M5 (5 months).

The grain moisture content varied significantly (*p* < 0.05) with treatments and storage duration. The onset of an increase in the moisture content of grains commenced in the second month for most treatments except for the T3 and T4 (Figure 4). T3 treated samples maintained a significantly steady lower moisture content, and this was not different across T4 samples throughout the storage period (Figure 4) compared with T1 which recorded an increase in moisture over the storage time. The significant variation in grain moisture content across different treatments and storage durations, with a notable synergistic effect, shows the fundamental role of storage bags in maintaining optimal moisture levels in stored grains.

T3 and T4 samples revealed an effectively steady moisture content throughout the storage period. This result aligns with the findings of Baoua et al. [36], who demonstrated that hermetic storage systems are highly effective at maintaining consistent moisture levels by creating an environment that limits moisture exchange with the external environment. The constant increase in moisture content observed in the T1 samples over the 5‐month storage time highlights the vulnerabilities associated with not using storage bags of quality functional material characteristics during storage. Moisture gain in grains is an important issue, as it can lead to mould growth, aflatoxin contamination and accelerated deterioration. Tefera et al. [39] found that grains stored in nonhermetic packaging, like the control treated sample, are prone to absorbing moisture from the surrounding environment, particularly in humid climates. This moisture accumulation not only affected the grain′s quality but also increased the risk of postharvest losses. Therefore, without proper storage, moisture content could rise significantly, compromising grain safety and marketability.

#### 3.2.2. Discolouration

**Figure 5:**
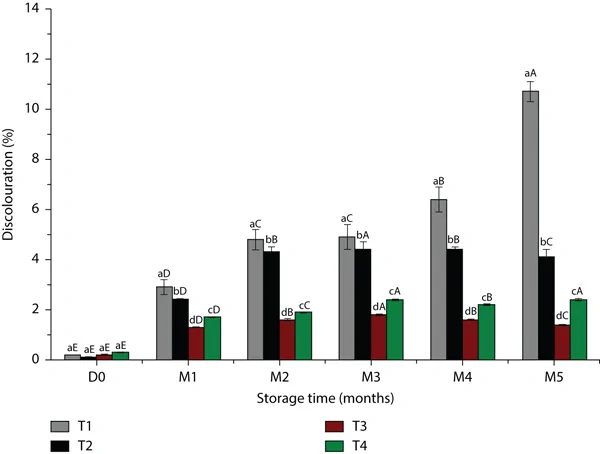
Observed impact of postharvest storage bags on maize discolouration during 5 months of storage. Values are means ± standard deviation (*n* = 3); Different lowercase letters indicate significant differences (*p* < 0.05) across the treatment (T1–T4), whereas different upper letters indicate significant differences (*p* < 0.05) across the storage time (D0‐M5)—based on Tukey′s test. T1 (control); T2 (jute bag); T3 (hermetic bag); T4 (nylon sack); D0 (Day 1); M1 (1 month); M2 (2 months); M3 (3 months); M4 (4 months) and M5 (5 months).

The results revealed a significant difference (*p* < 0.05) in the percentage of grain discolouration across treatments (T1, T2, T3 and T4) and storage durations (months) (Figure 5). At Day 1, all samples exhibited white grain colour. However, as storage time increased, discolouration, characterised by dark and brown spots became evident in all treatments T1 and T2, except T3 and T4 samples. Grain discolouration remained significantly lower (*p* < 0.05) in both T3 and T4 treated samples, in contrast to the T1 samples, which exhibited the highest discolouration levels.

The level of grain discolouration across the various treatments and storage periods highlights the critical role of packages in preserving grain quality during storage. According to Likhayo et al. [40], discolouration is a standard quality degradation marker in stored grains, often resulting from exposure to unfavourable storage conditions, including moisture and pest infestations. T3, which exhibited the lowest and most consistent levels of grain discolouration, is recognised for its superior ability to maintain grain quality over extended periods. Baoua et al. [36] demonstrated that hermetic storage effectively prevents grain discolouration by creating a sealed environment that reduces oxygen levels, thereby inhibiting the oxidative processes and pest activities that contribute to discolouration. The functionality of T3 in this study aligns with these findings, reinforcing its role as an essential tool for preserving grain colour and overall quality during long‐term storage. Although the T4 samples were less effective than T3 treatments, they offered better protection against discolouration compared with T2 treatments, suggesting that the material properties of these storage bags play a significant role in mitigating quality loss. Furthermore, the high levels of discolouration observed in T2 and T1 treatments could be due to the high moisture permeability of the packaging materials, allowing environmental humidity to penetrate and creating conditions favourable for mould and fungal growth. Yasin et al. [41] revealed that jute sacks are less effective at protecting against environmental factors such as humidity and pests, which are major contributors to grain discolouration. According to this observation, discolouration will probably worsen when storage conditions worsen, especially in nonhermetic maize storage situations.

#### 3.2.3. Grain Weight Loss

**Figure 6:**
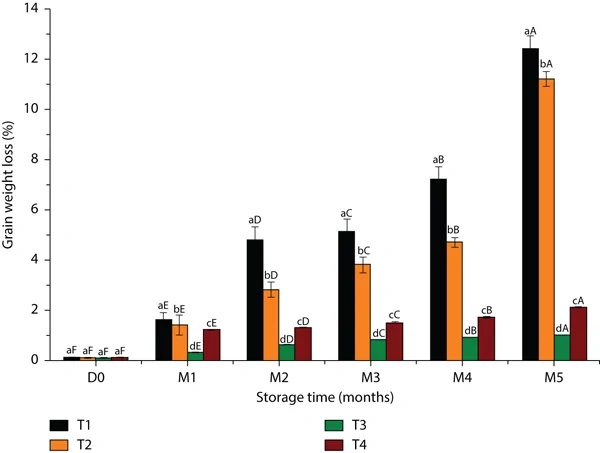
Observed impact of postharvest storage bags on maize grain weight during 5 months of storage. Values are means ± standard deviation (*n* = 3); different lowercase letters indicate significant differences (*p* < 0.05) across the treatment (T1–T4), whereas different upper letters indicate significant differences (*p* < 0.05) across the storage time (D0‐M5)—based on Tukey′s test. T1 (control); T2 (jute bag); T3 (hermetic bag); T4 (nylon sack); D0 (day 1); M1 (1 month); M2 (2 months); M3 (3 months); M4 (4 months) and M5 (5 months).

Figure 6 shows a significant (*p* < 0.05) observed impact across treatments and storage duration on maize grain weight loss. As storage time increased, grain weight loss also progressively rose. However, grain weight loss remained significantly lower and more stable in T3 and T4 treated samples. In contrast, the T1 sample indicates the highest grain weight loss after 5 months of storage. Overall, the mean grain weight loss increased in relation to storage period. These significant differences highlight the crucial role of both treatment and storage duration in maintaining grain quality and minimising weight loss.

The increase in grain weight loss over time aligns with the findings of Dubey et al. [42], who reported that prolonged storage periods exacerbate weight loss due to ongoing insect infestations and moisture loss. This trend was significant in traditional packaging choices, where inadequate protection often leads to substantial losses, which confirms the findings in this study that the choice of packages remains critical in mitigating these effects. T3, which showed significantly lower grain weight loss compared with other treatments (T1, T2 and T4), could be associated with the possibilities for an effective preservation of grain quality during storage. In addition, the lower grain weight loss observed in T4 compared with T2 further supports the notion that the material composition and barrier properties of the storage bags play a crucial role in grain preservation. The study′s findings also highlight the limitations of traditional packages, such as jute sacks, which were less effective in preventing grain weight loss compared with more advanced options like hermetic and nylon bags. Okori et al. [43] emphasised that integrating improved storage technologies, such as hermetic bags, can significantly reduce PHLs in maize compared with conventional storage technologies.

### 3.3. The Effect of Postharvest Storage Bags on the Nutritional Quality of Maize

#### 3.3.1. Protein Content

**Figure 7:**
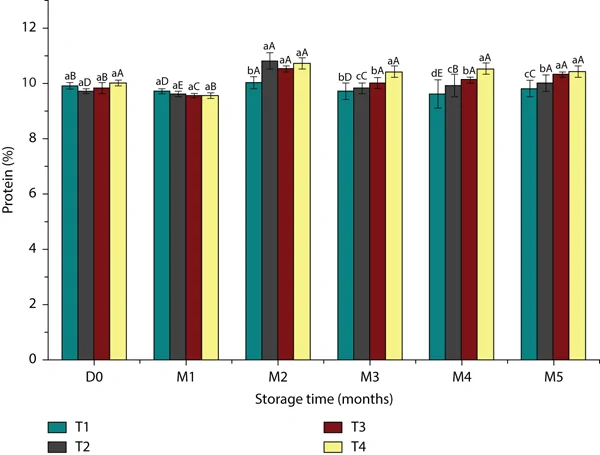
Observed impact of postharvest storage bags on maize protein during 5 months of storage. Values are means ± standard deviation (*n* = 3); different lowercase letters indicate significant differences (*p* < 0.05) across the treatment (T1–T4), whereas different upper letters indicate significant differences (*p* < 0.05) across the storage time (D0‐M5)—based on Tukey′s test. T1 (control); T2 (jute bag); T3 (hermetic bag); T4 (nylon sack); D0 (day 1); M1 (1 month); M2 (2 months); M3 (3 months); M4 (4 months) and M5 (5 months).

A significant difference (*p* < 0.05) in protein content was observed across the treatments and storage durations. Protein levels remained stable from Day 1 through the first (1) month but declined progressively in the second (2) month (Figure 7). The decline was more pronounced in the T1 treatment compared with the T2 treated samples, which showed a gradual decrease that did not differ significantly from T3 and T4 samples in the third (3) month. After 5 months of storage, T4 samples maintained the highest protein content yet, with no apparent statistical difference from the T3 samples. Overall, protein content showed a consistent decline among T1 samples relative to increasing storage duration.

In a study by Pathania et al. [44], it was reported that protein degradation in grains seems common during storage due to biochemical changes and environmental factors such as temperature and humidity. This study′s results indicate that protein loss was mitigated in grains stored in hermetic bags, which showed significantly lower decreases in protein content compared with the control, suggesting that hermetic storage is effective in preserving grain nutritional quality over time. Interestingly, the protein content in grains stored in nylon and jute sacks initially increased in the second month before declining in the subsequent months. This temporary increase might be explained by the natural variability in grain metabolism or slight moisture fluctuations that can cause transient changes in protein levels. Lutz and Coradi [45] observed similar trends in stored grains, noting that certain storage conditions result in short‐term increases in measurable protein content due to shifts in moisture content or the concentration of nitrogenous compounds. However, in this study, the gains were not sustained, and protein content eventually declined, particularly in the jute sacks, which exhibited less protection mechanism of the grains against environmental factors. Overall, hermetic and nylon bags were more effective in preserving the protein content of grains over the storage period compared with jute sacks and the controls. Namusalisi [46] emphasised the importance of using advanced storage technologies like hermetic bags to maintain grain quality, including protein content, by creating a controlled environment that limits exposure to oxygen, pests and moisture. This conclusion is consistent with this study′s findings, which show that hermetic bags provided superior protection against protein degradation, reinforcing the value of these technologies in reducing nutritional losses in stored grains.

#### 3.3.2. Crude Fibre Content

**Figure 8:**
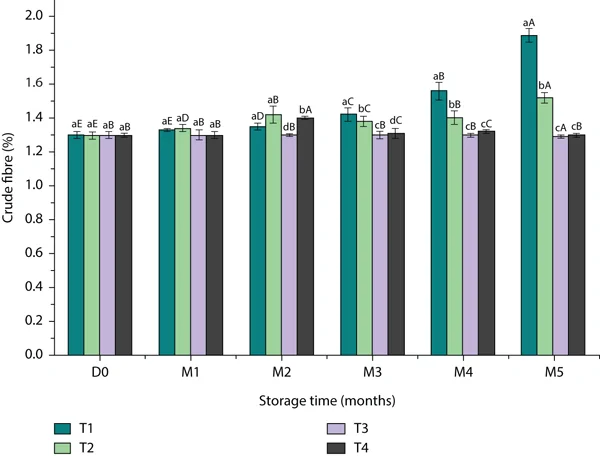
Observed impact of postharvest storage bags on maize crude fibre during 5 months of storage. Values are means ± standard deviation (*n* = 3); different lowercase letters indicate significant differences (*p* < 0.05) across the treatment (T1–T4), whereas different upper letters indicate significant differences (*p* < 0.05) across the storage time (D0‐M5)—based on Tukey′s test. T1 (control); T2 (jute bag); T3 (hermetic bag); T4 (nylon sack); D0 (day 1); M1 (1 month); M2 (2 months); M3 (3 months); M4 (4 months) and M5 (5 months).

The fibre content of T3 treated samples remained consistently low from Day 1 through to the 5‐month storage period, with no significant difference compared with T4 samples (M4 and M5). In contrast, T1 and T2 samples showed a gradual increase in fibre content from the second to the fifth month of storage (Figure 8).

Throughout the storage period, there were variations in the fibre contents for all treatments, particularly between Months 1 and 3, which may reflect natural variations in moisture content or metabolic changes within the grain. Similar fluctuations were observed by Deepa and Hebbar [47], who noted that fibre content in stored maize flour could vary due to factors like moisture absorption and enzymatic activity. The stability of fibre content in hermetic bags, which maintained low variability compared with other treatments, suggests that these bags are effective in mitigating the factors that contribute to fibre degradation or fluctuation. The pronounced changes in fibre content observed in the 4th and 5th months, especially in grains stored in jute sacks and the control, show the vulnerability of these packaging materials to environmental factors that can alter grain composition. The significant increases in fibre content in T1 and T2 treated samples may be attributed to the breakdown of other components like starches, leading to a relative increase in fibre proportion. Overall, the results demonstrate that hermetic and nylon bags were more effective in preserving the fibre content of grains compared with jute sacks and the control. The superior performance of hermetic bags can be attributed to their ability to create a stable, low‐oxygen environment that reduces the activity of microorganisms and pests, thereby minimising the degradation of fibre and other grain components. On the other hand, the higher increases in fibre content in jute and control treatments further point to the limitations of different forms of postharvest storage packaging materials.

#### 3.3.3. Carbohydrate Content

**Figure 9:**
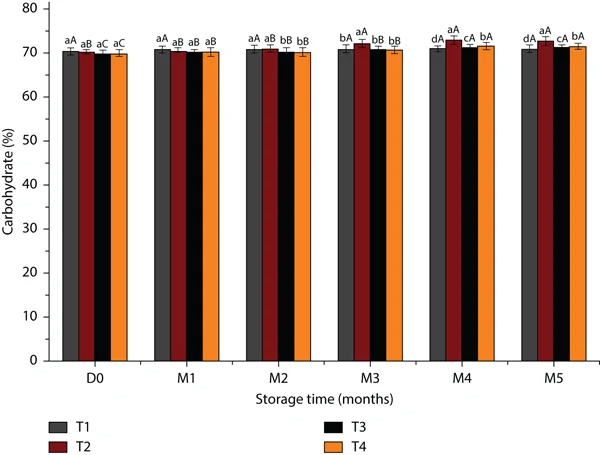
Observed impact of postharvest storage bags on maize carbohydrate during 5 months of storage. Values are mean ± standard deviation (*n* = 3); different lowercase letters indicate significant differences (*p* < 0.05) across the treatment (T1–T4), whereas different upper letters indicate significant differences (*p* < 0.05) across the storage time (D0‐M5)—based on Tukey′s test. T1 (control); T2 (jute bag); T3 (hermetic bag); T4 (nylon sack); D0 (day 1); M1 (1 month); M2 (2 months); M3 (3 months); M4 (4 months) and M5 (5 months).

In T2, carbohydrate levels stayed consistently low from Day 1 to Month 2, followed by a notable increase compared with T1, which saw a decline in carbohydrate content from Month 3 through the end of the 5‐month storage period. T3 samples maintained stable carbohydrate levels until 4th and 5th months, showing a statistical difference compared with T4 samples (Figure 9). At the end of the 5‐month storage, T2 samples exhibited the highest carbohydrate content, whereas T1 recorded the lowest.

The relatively stable carbohydrate content observed from Months 1–2 across all treatments suggests that the initial storage periods may not significantly affect carbohydrate levels. This stability is consistent with findings by Stathers et al. [48], who noted that carbohydrates in grains tend to be more resilient to short‐term storage effects compared with other macronutrients like proteins and fats. However, as storage progressed, the carbohydrate content began to fluctuate more noticeably, particularly in grains stored in jute sacks and controls, indicating the influence of prolonged exposure to environmental factors. The increase in carbohydrate content in grains stored in jute sacks during the 4th and 5th months may be attributed to the relative concentration effect, where moisture loss or degradation of other components like proteins and fats increases the apparent carbohydrate percentage. Bonazzi and Dumoulin [49] observed similar patterns, where poor storage conditions led to the degradation of noncarbohydrate components (proteins, fats, fibre and ash), causing a relative increase in carbohydrate content. On the other hand, the decrease in carbohydrate content observed in the T1 in the 4th and 5th months could be due to the higher susceptibility of grains in the control to spoilage, enzymatic activity or microbial degradation, which often leads to the breakdown of carbohydrates into simpler sugars or other byproducts [50]. This decrease aligns with findings by Wendt and Zhao [51], who reported that uncontrolled storage conditions could lead to significant losses in carbohydrate content due to these degradative processes. The effectiveness of hermetic and nylon bags in maintaining stable carbohydrate levels over the storage period underlines the value of advanced storage technologies in preserving grain quality. The minimal changes in carbohydrate content observed in grains stored in these bags reflect their ability to create a controlled environment that limits exposure to moisture, oxygen and pests, thereby reducing the risk of carbohydrate degradation. Odjo et al. [18] highlighted the importance of hermetic storage in protecting against environmental factors that can lead to nutrient loss, particularly carbohydrates in Mexico. In contrast, the greater fluctuations in carbohydrate content observed in jute sacks emphasise the limitations of poor and below‐average packaging materials.

### 3.4. Aflatoxin Levels of Maize

**Figure 10:**
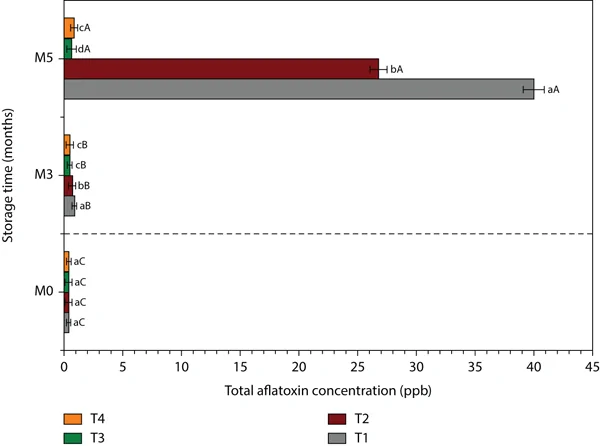
Observed impact of postharvest storage bags on maize aflatoxin level (parts per billion, ppb) during 5 months of storage. Values are means ± standard deviation (*n* = 3); Different lowercase letters indicate significant differences (*p* < 0.05) across the treatment (T1–T4), whereas different upper letters indicate significant differences (*p* < 0.05) across the storage time (D0‐M5)—based on Tukey′s test. T1 (control); T2 (jute bag); T3 (hermetic bag); T4 (nylon sack); D0 (Day 1); M1 (1 month); M2 (2 months); M3 (3 months); M4 (4 months) and M5 (5 months).

A significant (*p* < 0.05) relationship of treatment and storage duration on aflatoxin accumulation. Aflatoxin levels increased progressively over the 5‐month storage period (Figure 10). The T3 and T4 treatments maintained significantly lower and more stable aflatoxin levels compared with T1 and T2 samples. Notably, aflatoxin concentrations in T1 and T2 treatments exceeded Ghana′s acceptable limit of 15 ppb, whereas T3 and T4 samples remained below this permissible threshold.

This study found that hermetic bags effectively prevented aflatoxin accumulation in maize grain with a moisture content of 12%–13% over a 5‐month period. Results indicated that aflatoxin concentrations increased substantially in both jute bags and control samples by the third and fifth months, with the latter surpassing baseline levels of the former month. These treatments provided insufficient protection against the growth of toxigenic fungi during storage, surpassing Ghana′s regulatory threshold of 15 ppb (15 *μ*g/kg) at 5 months. On the other hand, grain kept in nylon sacks and hermetic bags kept aflatoxin levels well below starting points, maintaining safety and quality standards. According to Maina et al. [52], PICS bags effectively controlled the initial aflatoxin levels in maize stored for 3 months (< 5–42.7 ppb) with 55% lower than polypropylene (PP) bags. In contrast, Hell et al. [53] identified insect damage, storage for 3–5 months, and the use of *Khaya senegalensis* bark or other native plants as a storage protectant as the factors linked to greater aflatoxin contamination in maize in four agroecological zones of Benin, West Africa. Hence, the effective aflatoxin control observed in hermetic treatments is likely due to the consistently low moisture content maintained throughout the 5‐month study.

## 4. Conclusion

The association between postharvest quality characteristics of maize in the Tain District of the Bono Region, Ghana, and storage packaging methods, such as unpackaged corn, jute bags, hermetic bags and nylon sacks was investigated. The study demonstrates that storage bags significantly affect maize storage quality. However, hermetic bag proved superior functionalities, preventing insect damage, maintaining moisture, preserving protein content, minimising grain discolouration, reducing grain weight loss and controlling aflatoxin levels (6.71 ppb) relative to safe threshold permissible range of 15 ppb in Ghana. The nylon sack offered intermediate protection, whereas the jute sack and unpackaged control performed poorly, with unsafe aflatoxin levels (28.36–42.38 ppb) exceeding Ghana′s 15 ppb safety limit. These findings have critical implications for Ghana′s food security, where maize comprises 50%–60% of cereal production. The research strongly recommends hermetic storage adoption, with nylon sacks as an economical alternative to traditional jute packaging. However, future research should create affordable storage materials that local farmers can use while also improving the safety and quality of the maize and addressing sustainability issues with environmentally friendly storage materials. To reiterate the trade‐off between hermetic storage′s expenses and advantages, not all farmers may find hermetic storage to be beneficial due to budgetary constraints. It is therefore very essential to investigate local farmers′ attitudes and perceptions regarding the use of hermetic bags and other contemporary storage methods and how this affects the economic gain from maize in the supply chain.

## Author Contributions

K.D.: conceptualization, methodology, investigation, writing—original draft, data curation, formal analysis and resources. P.K.B.: conceptualization, methodology, writing—review and editing, validation, supervision and project administration. E.N.A.: data curation, methodology, validation, writing—original draft and investigation. E.F.B.: data curation, software, methodology, formal analysis, investigation and writing—review and editing.

## Funding

No funding was received for this manuscript.

## Conflicts of Interest

The authors declare no conflicts of interest.

## Data Availability

The data that support the findings of this study are available from the corresponding authors upon reasonable request.
